# The automated Greulich and Pyle: a coming-of-age for segmental methods?

**DOI:** 10.3389/frai.2024.1326488

**Published:** 2024-03-12

**Authors:** Rashmi Chapke, Shruti Mondkar, Chirantap Oza, Vaman Khadilkar, Tim R. J. Aeppli, Lars Sävendahl, Neha Kajale, Dipali Ladkat, Anuradha Khadilkar, Pranay Goel

**Affiliations:** ^1^Department of Biology, Indian Institute of Science Education and Research Pune, Pune, India; ^2^Hirabai Cowasji Jehangir Medical Research Institute, Pune, India; ^3^Department of Health Sciences, Savitribai Phule Pune University, Pune, India; ^4^Jehangir Hospital, Pune, India; ^5^Division of Pediatric Endocrinology, Department of Women's and Children's Health, Karolinska Institutet, Stockholm, Sweden

**Keywords:** bone aging, Greulich and Pyle (GP), computer vision, RSNA image share, personalizability

## Abstract

The well-known Greulich and Pyle (GP) method of bone age assessment (BAA) relies on comparing a hand X-ray against templates of discrete maturity classes collected in an atlas. Automated methods have recently shown great success with BAA, especially using deep learning. In this perspective, we first review the success and limitations of various automated BAA methods. We then offer a novel hypothesis: When networks predict bone age that is not aligned with a GP reference class, it is not simply statistical error (although there is that as well); they are picking up nuances in the hand X-ray that lie “outside that class.” In other words, trained networks predict *distributions* around classes. This raises a natural question: How can we further understand the reasons for a prediction to deviate from the nominal class age? We claim that *segmental aging*, that is, ratings based on characteristic bone groups can be used to qualify predictions. This so-called *segmental GP method* has excellent properties: It can not only help identify differential maturity in the hand but also provide a systematic way to extend the use of the current GP atlas to various other populations.

## 1 Introduction

The Greulich and Pyle (GP) method of bone age rating (Greulich and Pyle, 1959) has a considerable history of use in not only manual rating but also automated analysis of X-rays. GP relies on “matching” a given X-ray against a reference X-rays of different ages; these templates comprise the GP atlas. Early attempts at automated systems included semi-automated methods, such as HANDX (Michael and Nelson, 1989), and fully-automated ones (Pietka et al., 1991; Hill and Pynsent, 1994; Sato et al., 1999). Bone age assessment (BAA) has recently been greatly advanced by the availability of the Radiological Society of North America (RSNA) dataset, which consists of a large number of X-rays which have been rated manually by multiple raters. The 2017 RSNA Pediatric Bone Age Machine Learning Challenge (Halabi et al., 2019) stimulated several deep learning methods, which have shown great success on the RSNA data (Chu et al., 2018; Larson et al., 2018; Halabi et al., 2019; Siegel, 2019; Wu et al., 2019; Alblwi et al., 2021; Liu et al., 2022; Beheshtian et al., 2023). In terms of performance, the RSNA challenge reported mean absolute deviation (MAD) >4.27 months, with a median of 5.99 months, among 48 submissions (Halabi et al., 2019). This question continues to attract attention, in part, in an attempt to improve the prediction accuracy of networks.

### 1.1 The segmental decomposition of the hand

One approach is to seek improvements using better networks and computations. On the other hand, a number of authors have begun to extend the GP method itself beyond its classical presentation.

A particularly fruitful point of view is to divide the hand into functional groups of bones, and we term these hand “segments.” For instance, the radius, ulna, the carpals, and short bones (metacarpals and phalanges) can be considered as important hand segments (Oza et al., 2023). The idea behind such a decomposition of the hand into segments is to group bones that are likely to grow uniformly together, and importantly, observe any inter-segmental differences in maturity. Bone age is determined not only from the full hand but also taking into account segmental age differences, if any. That is, segments are *independently* rated and compared with the whole hand rating.

**Figure 1:**
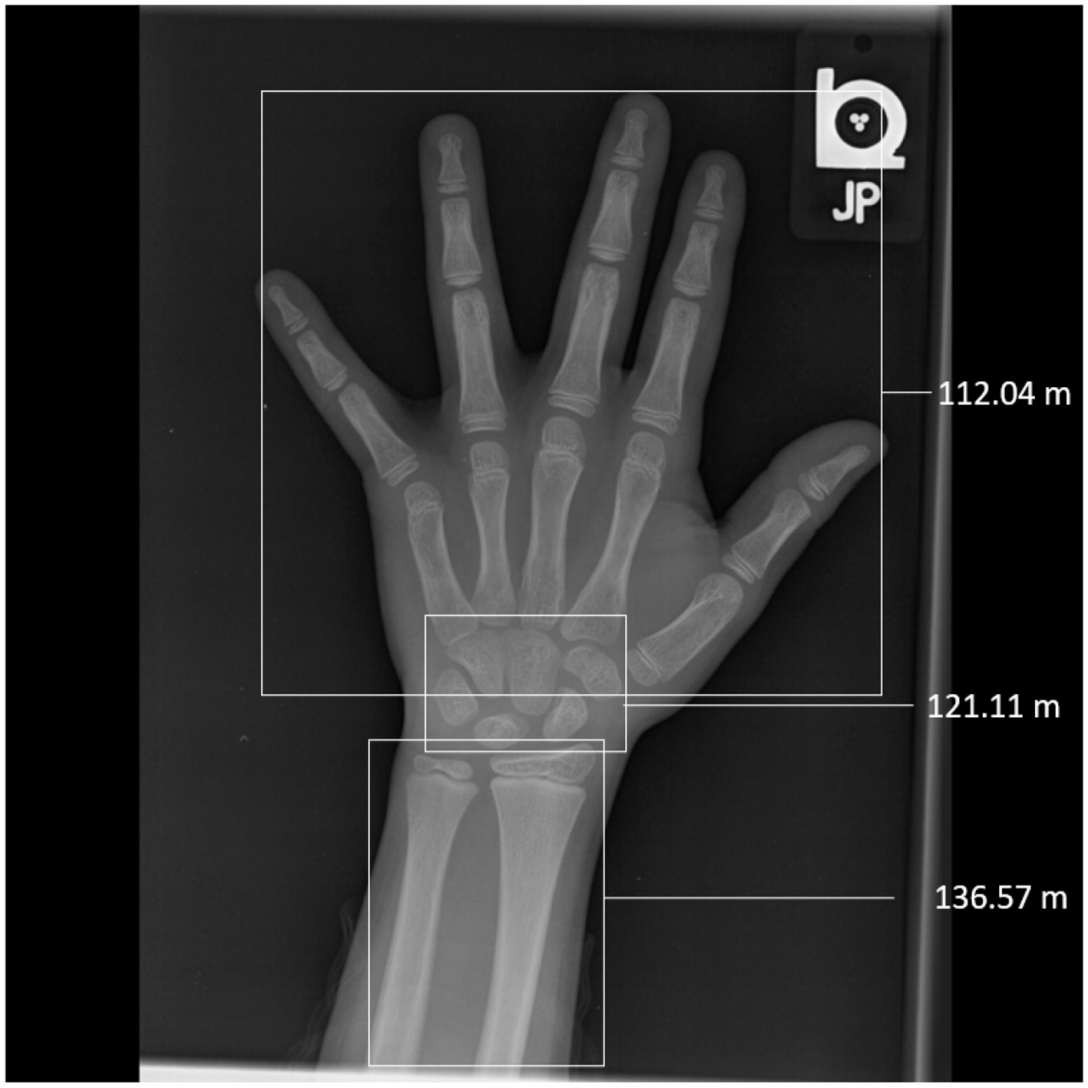
Segmental rating on the RSNA image, 1547.png, a male hand rated to be 10.5 years (126 months) of age. Network prediction using the full hand image was 10.2 years (122.6 months). Each segment is boxed, and its predicted age is marked alongside. Notice the discrepancy in the segmental predictions—between 112 and 136 months—suggestive of a marked differential maturity in the hand. In Oza et al. (2023), manual segmental ratings were: short bones: 10.5 years (126 months); radius and ulna: 10 years (120 months); carpals: 10 years (120 months). Notice the carpals prediction matches the manual rating, the short bones are somewhat underestimated by the network, and the radius and ulna are relatively advanced.

A number of studies have attempted to rate not only the full hand but also segments (Simu and Lal, 2017; Chapke, 2023; Jung et al., 2023; Oza et al., 2023). The motivation in the study mentioned in the reference Simu and Lal (2017) and Jung et al. (2023) appears to be largely computational, that is, to extract relevant portions of the image from an irrelevant background. Our analysis in this article follows the study mentioned in the reference Chapke (2023) closely; please see further details therein. Figure 1 illustrates automated segmental rating. Chapke (2023) describes a DenseNet architecture that combines segmental and full-hand predictions to obtain an MAD for 6.7–6.9 months for boys and 7.4–7.6 months for girls. Jung et al. (2023) employ a different segmentation: phalanges, metacarpals, and carpals using an Xception model that they obtain an ensemble MAD of 5.69.

We argue here that the methodology of segmental GP runs much deeper than computational concerns alone. While segments do provide independent assesments of BAA, they can also be used to identify when (and why) a hand is likely to deviate from the reference atlas. When the bones of the hand are differentially matured, a classical GP rating is difficult to apply. Here, we will show that the segmental GP rating is particularly useful in such a scenario. For instance, in the study mentioned in the reference, Oza et al. (2023) we show that the hand segments contribute *different weights* to an overall age assessment; a more accurate rating is obtained when this is taken into account.

We, therefore, present a novel perspective comparing these two lines of reasoning. We proceed first from a careful assessment of the achievements on the RSNA data so far. Our discussion hinges on the question of rater variability relative to the BAA and how it can be mitigated. In particular, we suggest that the methods employed hitherto are already close to the limits achievable for lowering MAD. Next, we show that it is possible to use segmental GP methods to analyze network predictions in greater detail.

### 1.2 Can computer vision unambiguously remove rater variability?

#### 1.2.1 Is automating GP a classification or regression problem?

In the GP framework, an independent rater always assigns a bone age corresponding to one of the reference classes. Naively, one might therefore be tempted to say that BAA machine learning is a *classification* problem. On the other hand, when two or more raters age an X-ray, they often differ in their assessment. A common way of accounting for this “inter-rater variability” is to average over multiple ratings. This effectively introduces an uncertainty in the age assessment, in the sense that the final, averaged age rating may not coincide with *any* of the reference GP classes. For instance, suppose it is ambiguous which of two nearby reference ages, 120 or 132 months, should an X-ray be assigned to? Each rater is expected to rate an X-ray to one of these classes; however, due to inter-rater disagreement, an average rating will lie *between* 120 and 132, say, 126 months. Correspondingly, the ground truth label during machine learning training is 126 months. Training on such labels is expected to recover this intermediate, average rating. BAA machine learning has hitherto been treated as a *regression* problem; the training objective is to obtain the smallest MAD between predicted and true labels over the entire training dataset.

#### 1.2.2 Interpreting machine learned BAA

In light of the above discussion, it is important to reflect on what is the underlying expectation from an automated BAA system? There are two possibilities.

On one hand, averaging is an excellent means of overcoming rater variability. That is, if given sufficient raters, idiosyncratic variation is reasonably well accounted for. Multiple human ratings are, however, difficult to obtain in practice. It is thus attractive to *supplement clinical ratings with automated, AI methods of prediction*.

On the other hand, a natural question is to ask if “objective” methods—such as machine learning and computer vision—can give us an answer which disambiguates between raters? In other words, can automated bone aging *overcome* inter-rater variability? That is, can one find a genuine, “true” label that is discovered in some objective manner?

While the former expectation is reasonable, the latter cannot be satisfied. In other words, *regression-based BAA is better thought of as an attempt to reproduce what multiple human raters would come up with rather than adjudicating among discrepant ratings*. In fact, we show below that some of the best performing AI methods—the so-called ensembles-of-networks—are themselves constructed as averages over multiple (model) ratings.

#### 1.2.3 Regression for optimal MAD

The reason why regression using MAD as a loss metric cannot resolve rater variability has to do not only with the way in which multiple machine learning models are combined toward a prediction but also with the physiology of bone development itself. The following simple argument illustrates this paradox.

Any trained automated system will have learnt to reproduce a rater-averaged bone age; this does not *resolve* rater discrepancy, it *reproduces* it. On the other hand, suppose that we expect the trained algorithm to pick out *one* of 120 or 132 months, regardless of the specific prediction choice, MAD would be 6 months (since the ground truth label is 126). In other words, a machine learning system trained to reduce loss (on “noisy labels,” as it were) is faced with two incompatible choices: either rater variation is embedded into the solution by design—which, in turn, throws the baby out with the bathwater—or we are forced to accommodate some *minimum* MAD, which represents the difference between the machine prediction and an (ideal) reference rating.

Such a threshold can be obtained on theoretical grounds by estimating a Bayes decision boundary with respect to the reference ages in the respective GP atlases. It is expected to be 3.8 months in boys and 4.2 months in girls for the standard GP atlas; see Supplementary Data for details. Regression learning cannot be expected to reduce MAD much lower than this minimum.

#### 1.2.4 Modeling via ensembles

A well-known technique employed in training deep learning models is to use *ensembles of models* in prediction. Ensembles are essentially *different models trained on the same data*. There are numerous techniques for creating ensemble models, utilizing not only different network architectures and their hyperparameters but also multiple partitions of the data or combinations thereof. Each of the ensemble models is used to provide their respective predictions on (new, test) data which are averaged together into a joint prediction. Ensembling works by reducing the variance of the final output. It is thus possible to reduce MAD using ensemble learning by training multiple networks on the data and considering the ensemble MAD; this can be expected to be somewhat lower than the MAD of (any of) the contributing networks. Ensemble rating is thus best interpreted as a committee of AI raters (networks), some of which are likely to predict one class, and others are likely to predict a nearby class.

**Figure 2:**
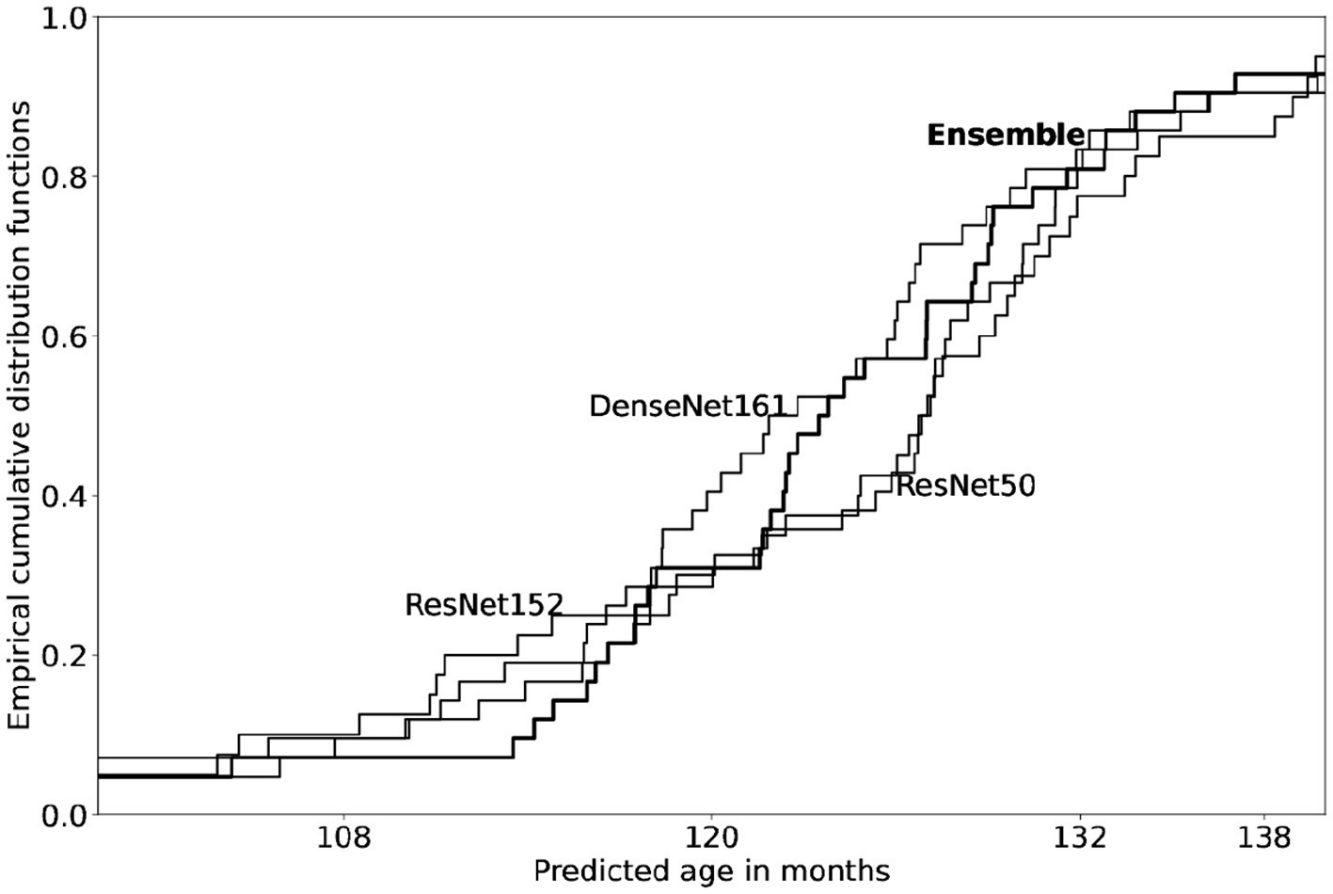
Empirical cumulative distribution functions for bone age predicted from three models on all validation X-rays of age 120 months (boys). Model architectures include two ResNet's (50 and 152) and one DenseNet161, each trained on the full RSNA training data (sex: male); see Chapke (2023) for the general methodology of model construction. Note that each model is evaluated on validation set of X-rays labeled with age 120 months (*n* = 42). Among the three models, predictions from each network differ slightly in estimates of age. The ensemble ecdf is overlaid in bold; ensemble predictions are observed to align with the average of the three models.

In our example above, manual rating was expected to place the X-ray between 120 and 132. Figure 2 shows *how three separate networks, each trained on the full RSNA data, perform on all the (validation) X-rays of male children aged 120 months*. Notice that each of the networks mark the X-rays to somewhat different ages, as evidenced by the difference in their corresponding empirical cumulative distribution functions (ecdf's). The predicted age probability density is centered roughly close to 120 months, as is to be expected. More to the point, the predicted ages *range between 108 and 132 months*. In other words, predictions are more or less centered on the correct (ground label) age class but range between one class below and above. This distribution is a central feature of all models and their predictions. Each network of an ensemble effectively specializes on different domains of the data; the resultant network average is a rating with low variance. In other words, the ensemble average is a sober, *reliable* estimate: The collective decision of a number of raters is a *consensus* score. *Ensemble network rating does not solve a disambiguation problem, as one might have initially assumed; instead it simulates multiple rating*.

Pan et al. (2019) have considered ensembling of 48 heterogeneous models of 2017 RSNA Pediatric Bone Age Machine Learning Challenge. The authors explored ensembles comprising up to 10 models and observed that ensembles with less correlation between the models yielded better results when evaluated using MAD. They also noted that combining the best individual models produced slightly better outcomes compared with searching through all feasible ensembles. The ensembling of machine learning models led to an improvement in the generalization error from 4.55 to 3.79 months, surpassing the performance of a single model. As we note above, these numbers are already close to the theoretical estimates, thus *machine learning models trained on RSNA data cannot be expected to improve MAD much lower*.

### 1.3 The segmental and ensemble methods are different strategies

The preceding analysis illustrates a uniform cohort of data, such as images of all labeled 120 months of age, when looked through the prism of deep learning models refracts them along a *continuum*. Images that are otherwise considered *equivalent in the sense that they all belong to the same class* do have subtle variations; it is quite possible that machine learning is picking up on these. After all, one can be readily convinced that Greulich and Pyle attempts to meaningfully *discretize* the maturity progression of the hand: Images are spread out in distributions centered on reference ages, and a label such as 120 is merely a convenience denoting the central tendency of that density.

The segmental method approaches this problem differently. It recognizes that the important equation to answer is not only what the “right” label for an image is. Instead, it asks: “What are the variations in a hand that contribute to an average rating, and which are features that *deviate* from the mean?” In the Figure 1 considered above, network rating was predicted to be 122.57 months for an image with GP rating of 126 months; notice, however, that the segment-wise predictions all differ. This can thus be interpreted as saying that the hand contains maturity features that have a central tendency lying between the GP reference plates at 120 and 132 months. Closer (segmental) examination reveals the degree to which bones are differentially advanced relative to that reference.

#### 1.3.1 The concordance curve

We now introduce some useful terminologies that make it considerably easier to discuss the accuracy of network predictions.

Most images in the RSNA dataset align with one or the other GP reference class, we call these “concordant” images. There are some images, however, that do not align, and this constitutes the “discordant” set. The RSNA training set consists of 6,833 male and 5,778 female radiographs; among the male files, there are 5,909 concordant 924 discordant files, while the female class contains 4,882 concordant images and 896 discordant images.

Suppose that a network prediction needs to select among discrete classes, such as 108, 120, and 132; this can be done based on their relative closeness. Network predictions of a GP class (on, say, images concordant with 120 months) can be described by the corresponding ecdf, as shown in Figure 2. We term this the “concordance curve” of that class. The position of a new prediction on this curve enables us to decide whether is 120 a good label for this image. A clinician may decide, for example, that a deviation within one standard deviation of 120 is not large enough to probe further. On the other hand, when the discordance is large, it might be helpful to ask: What is the underlying cause in terms of a differential maturity of the hand?

## 2 Discussion and outlook

In this perspective, we have argued an intriguing, attractive property of machine learning that it reproduces BAA *distributions*. While the initial RSNA challenge was motivated by a reduction in MAD (Halabi et al., 2019) *over the population*, any application to the *individual* suffers from the same statistical caveats that all epidemiological studies do. It is fortuitous that convolutional neural networks pick up on a rich feature set embedded in the X-ray. Thus, when network predictions on two X-rays with the same GP label differ, this is not simply a chance occurrence; there is reasoning hidden deep in the feature set of the hand that the network is able to tell the two apart. The segmental GP method is an attempt to exploit this feature set fully. The novel tool we introduce for this purpose is the concordance curve. When two concordant X-rays differ in prediction, they are first identified by their location on the concordance curve. Next, segmental ratings are obtained, and these reveal the underlying differences in maturity in the hand. In this manner, automated BAA is a much more general methodology, capable of telescoping down to far greater resolution than ordinary GP.

Thus, at its simplest, the segmental GP is an excellent ensemble method (Jung et al., 2023), especially since each of the hand segments represent uncorrelated data. It is, however, much more than that: it is GP adapted to reveal maturity differences in different parts of a hand. This provides a vocabulary for raters that might disagree in their overall age assessment, to compare what are the specific locations they do (not) agree on. We claim that this places the clinician firmly in control of decision processes. “Interpretable AI” has become an emergent challenge that is often observed as threatening the adoption of digital health solutions. Segmental GP, by allowing the clinician to investigate the underlying rationale for a prediction, thus places them firmly “in-the-loop.” We believe that this will greatly improve confidence in the technology.

We are confident that the segmental GP method is the new way forward for automated, AI-based BAA.

## Data availability statement

Publicly available datasets were analyzed in this study. The RSNA Pediatric Bone Age Challenge image datasets and annotation files can be downloaded from https://www.rsna.org/education/ai-resources-and-training/ai-image-challenge/RSNA-Pediatric-Bone-Age-Challenge-2017.

## Ethics statement

The studies involving humans were approved by The Institutional Ethics Committee, HCJMRI (August 2019), and since de-identified data were used a waiver was granted by the Ethics Committee (July 2020). This study was approved by the Ethics Committee of the Indian Institute of Science Education and Research Pune (IHEC/Admin/2021/014). A data sharing agreement was signed between HCJMRI and IISER Pune and KI for collaborative work (May 2021). The studies were conducted in accordance with the local legislation and institutional requirements. Written informed consent for participation in this study was provided by the participants' legal guardians/next of kin.

## Author contributions

RC: Formal analysis, Software, Visualization, Writing—review & editing. SM: Data curation, Investigation, Methodology, Writing—review & editing. CO: Data curation, Investigation, Methodology, Writing—review & editing. VK: Investigation, Methodology, Writing—review & editing. TA: Investigation, Methodology, Writing—review & editing. LS: Conceptualization, Funding acquisition, Investigation, Methodology, Project administration, Supervision, Writing—review & editing. NK: Investigation, Writing—review & editing. DL: Investigation, Project administration, Writing—review & editing. AK: Conceptualization, Data curation, Funding acquisition, Investigation, Methodology, Project administration, Writing—review & editing. PG: Conceptualization, Formal analysis, Funding acquisition, Investigation, Methodology, Project administration, Supervision, Writing—original draft, Writing—review & editing.
