# Supplementary material for: The automated Greulich and Pyle: a coming-of-age for segmental methods?
Source: Front Artif Intell. 2024 Mar 12;7:1326488. doi: 10.3389/frai.2024.1326488 (PMC10963464; doi:10.3389/frai.2024.1326488)
Supplement: Supplementary file 1 [file Data_Sheet_1.pdf]

Supplementary Material

Chapke et al.

1 Deriving an estimate of minimum MAD

In this manuscript we have claimed that neural networks that are trained to minimize MAD with respect to ground truth inevitably suffer from a limitation, namely, there is a **minimum MAD attached with these predictions**. This arises from an essential ambiguity between one atlas class and its neighbor; see the main text for more details. A theoretical estimate of this minimum MAD can be obtained as follows.

The GP atlas includes a collection of 31 reference radiographs for boys and 28 for girls, spanning ages between 0 and 19 years. GP classes for boys are: 0, 3, 6, 9, 12, 15, 18, 24, 32, 36, 42, 48, 54, 60, 72, 84, 96, 108, 120, 132, 138, 150, 156, 162, 168, 180, 186, 192, 204, 216, 228 months. We argue that all images that are one-half class width apart on either side are considered *equivalent*. Thus all images half-way between 108 and 120 and half-way between 120 and 132 will all be marked to 120, that is, all images predicted to be between 114 and 126 are equivalent (to 120). Thus one computes an "inter-class distance (ICD)" between successive GP reference classes. The minimum MAD is roughly half this ICD. Thus, calculating the ICDs between the classes above shows that the ICD in the boys GP atlas is 7.6 months *on average*. **Thus the minimum MAD to be expected for boys is 3.8 months.**

Similarly, GP classes in the girls atlas are: 0, 3, 6, 9, 12, 15, 18, 24, 30, 36, 42, 50, 60, 69, 82, 94, 106, 120, 132, 144, 156, 162, 168, 180, 192, 204, 216 months. The average ICD for GP classes in girls is 8.3 months. **Thus the minimum MAD to be expected for boys is 4.2 months.**

Notice that this *estimate depends only on the atlas* and the reference classes specific to boys and girls. It is independent of the any particular machine learning method that might be used for prediction.

2 Example concordance curves

Fig. 1 shows the concordance curve (see main text for details) of two classes, 120 and 132 months. These are obtained in the following way. A DenseNet-161 model was trained on full-hand images of the full RSNA dataset. Only those validation set images were retained for which the ground truth label was *exactly* 120 (132) months. In other words, only "concordant" images were examined. This means that we are examining the prediction only for those images which

have been manually agreed to belong to class 120 months. The prediction of the network are examined to ask: "How does the network view these images?"

It is clear that the network sees in these images a distribution of ages roughly centered on 120. These predictions are depicted as an empirical cumulative distribution function (eCDF) in Fig. 1. The eCDF can be reasonably well fit by a gaussian CDF with the corresponding sample mean and standard deviation.

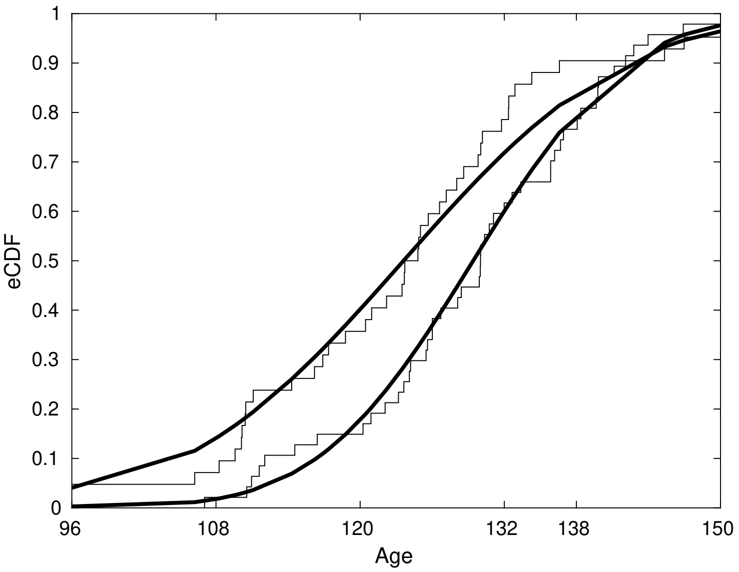

Figure 1: The concordance curves obtained on the RSNA validation dataset for ages 120 and 132 months (boys; n=42 and n=47, respectively). The empirial CDFs correspond to the network predictions. These are fitted to gaussian densities (smooth curves), with $\mu = 123.6$, $\sigma = 14.5$ and $\mu = 129.4$, $\sigma = 10.2$ respectively.

Note that these are concordant images according to the ground truth and that the networks have been trained to optimally reduce MAD as low as possible, on the entire data as a whole. In spite of this, we find that the **network predictions are (normally) distributed around their respective classes**. There is some bias as well, for instance the 120 (132) month curve is centered on 123.6 (129.4) months. We have claimed in the present article that regardless of the specific details of the learned networks, *all* deep networks have this feature.

We have previously proposed that the reason for this distributional spread is differential maturity in the hand [1]. In this manuscript we introduce the concordance curve as a means of gauging the extent of maturity variation via segmental ratings. It is clear from this figure that *the further away from the mean a prediction lies, the more likely it is to contain features of the neighboring class(es)*. Thus the variance attached with a concordance curve can be used to

assess differential maturity associated with a prediction.

3 Segmental ratings evaluated on RSNA validation dataset

We examined segmental ratings on the RSNA validation data. The question arises: If the network has been trained on only *a segment of the hand* along with the RSNA ground truth label, how well would it do? In other words, can an RSNA label – which reflects a manual average rating of evaluation of the full hand – be predicted from a segment of the hand? Figure 2 suggests that it can: Segment-wise predictions are very strongly correlated with the RSNA rating.

4 Comparison of manual and automatic predictions on the Oza et al. subset of the RSNA data

In [1] we had previously manually evaluated *segment-wise* bone ages for a subset of the RSNA data. This so-called Oza et al. subset included select X-rays that were distributed roughly uniformly across ages.

Figure 3 shows Bland-Altman plots of manual and network predictions. The associated variations are consistent with expectation. The good correspondence between the two methods demonstrates that the automated, network predictions are in line with manual rating for the large part. We also note, however, a few marked outliers which warrant further investigation; this is left for future studies.

We remark that the Oza et al. subset is from the RSNA *training* data. Since networks have, in principle, been exposed to this data during training, prediction error on this same data is expected to be low, roughly about 2-3 months. This implies that if there is a large discrepancy from the manual rating, that can effectively be attributed to the *manual rating differing substantively from the RSNA label.* In other words, a useful interpretation of these plots is that they suggest the extent to which each segment-wise rating could differ from the original RSNA label.

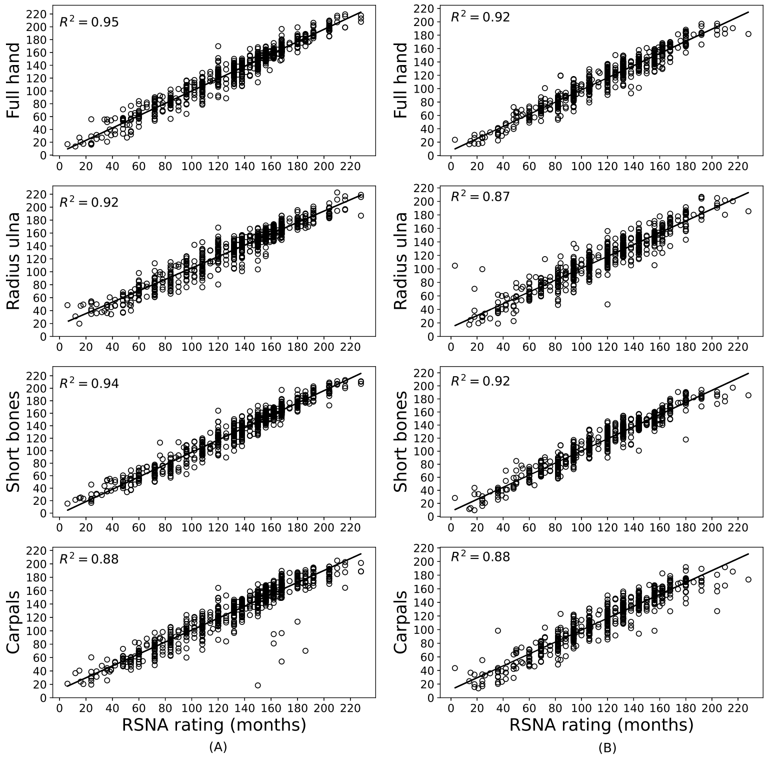

Figure 2: Effectiveness of the segmental bone age predictions (in months) on RSNA (A) boys (n=768) and (B) girls (n=647). Evaluations are carried on the RSNA validation set. Subfigures compare the age-predicted using models trained on short bones, carpals, and radius ulna, and full hand against RSNA ground truth labels.

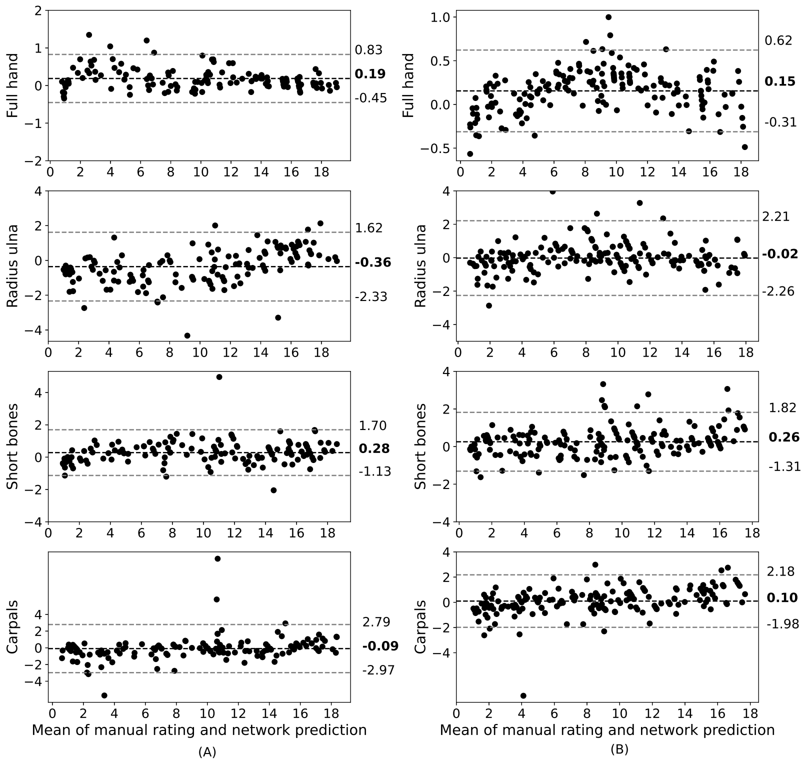

Figure 3: Bland-Altman plots for (A) boys (n=141) and (B) girls (n=178) for a subset of RSNA training data. These radiographs were rated as described in [1] for short bones, radius ulna, carpals, and full hand. The x-axis represents the mean of manual rating and network prediction in years, and the y-axis represents the difference between manual rating and network prediction; mean and standard deviations are depicted on the respective plots.

References

[1] Chirantap Oza, Anuradha V Khadilkar, Pranay Goel, Tim Aeppli, Shruti A Mondkar, Nikhil Shah, Nikhil Lohiya, Hemchand K Prasad, Prashant Patil, Neha A Kajale, Vaman Khadilkar, and Lars Lars Sävendahl. Standardization of weightage assigned to different segments of the hand X-ray for assessment of bone age by the Greulich Pyle method. *medRxiv*, 2023. doi:10.1101/2023.06.02.23290917.
